# *Escherichia coli* Heat-Stable Enterotoxin Mediates Na^+^/H^+^ Exchanger 4 Inhibition Involving cAMP in T_84_ Human Intestinal Epithelial Cells

**DOI:** 10.1371/journal.pone.0146042

**Published:** 2015-12-29

**Authors:** Ana R. Beltrán, Luciene R. Carraro-Lacroix, Camila N. A. Bezerra, Marcelo Cornejo, Katrina Norambuena, Fernando Toledo, Joaquín Araos, Fabián Pardo, Andrea Leiva, Carlos Sanhueza, Gerhard Malnic, Luis Sobrevia, Marco A. Ramírez

**Affiliations:** 1 Cellular Physiology Laboratory, Biomedical Department, Faculty of Health Sciences, Universidad de Antofagasta, Antofagasta 1270300, Chile; 2 Department of Education, Faculty of Education, Universidad de Antofagasta, Antofagasta 1270300, Chile; 3 Department of Physiology and Biophysics, Institute of Biomedical Sciences, University of São Paulo, São Paulo 3550308–1009, Brazil; 4 Department of Basic Sciences, Faculty of Sciences, Universidad del Bío-Bío, Chillán 3780000, Chile; 5 Department of Physiology, Faculty of Pharmacy, Universidad de Sevilla, Seville E-41012, Spain; 6 University of Queensland Centre for Clinical Research (UQCCR), Faculty of Medicine and Biomedical Sciences, University of Queensland, Herston, QLD 4029, Queensland, Australia; 7 Cellular and Molecular Physiology Laboratory (CMPL), Division of Obstetrics and Gynecology, Faculty of Medicine, School of Medicine, Pontificia Universidad Católica de Chile, Santiago 8330024, Chile; The University of Texas Health Science Center, UNITED STATES

## Abstract

The enterotoxigenic *Escherichia coli* strains lead to diarrhoea in humans due to heat-labile and heat-stable (STa) enterotoxins. STa increases Cl^-^release in intestinal cells, including the human colonic carcinoma T_84_ cell line, involving increased cGMP and membrane alkalization due to reduced Na^+^/H^+^ exchangers (NHEs) activity. Since NHEs modulate intracellular pH (pH_i_), and NHE1, NHE2, and NHE4 are expressed in T_84_ cells, we characterized the STa role as modulator of these exchangers. pH_i_ was assayed by the NH_4_Cl pulse technique and measured by fluorescence microscopy in BCECF–preloaded cells. pH_i_ recovery rate (*dpHi/dt*) was determined in the absence or presence of 0.25 μmol/L STa (30 minutes), 25 μmol/L HOE-694 (concentration inhibiting NHE1 and NHE2), 500 μmol/L sodium nitroprusside (SNP, spontaneous nitric oxide donor), 100 μmol/L dibutyryl cyclic GMP (db-cGMP), 100 nmol/L H89 (protein kinase A inhibitor), or 10 μmol/L forskolin (adenylyl cyclase activator). cGMP and cAMP were measured in cell extracts by radioimmunoassay, and buffering capacity (ß*i*) and H^+^ efflux (*J*_*H*_^+^) was determined. NHE4 protein abundance was determined by western blotting. STa and HOE-694 caused comparable reduction in *dpHi/dt* and *J*_*H*_^+^ (~63%), without altering basal pH_i_ (range 7.144–7.172). STa did not alter *ßi* value in a range of 1.6 pH_i_ units. The *dpHi/dt* and *J*_*H*_^+^ was almost abolished (~94% inhibition) by STa + HOE-694. STa effect was unaltered by db-cGMP or SNP. However, STa and forskolin increased cAMP level. STa–decreased *dpHi/dt* and *J*_*H*_^+^ was mimicked by forskolin, and STa + HOE-694 effect was abolished by H89. Thus, incubation of T_84_ cells with STa results in reduced NHE4 activity leading to a lower capacity of pH_i_ recovery requiring cAMP, but not cGMP. STa effect results in a causal phenomenon (STa/increased cAMP/increased PKA activity/reduced NHE4 activity) ending with intracellular acidification that could have consequences in the gastrointestinal cells function promoting human diarrhoea.

## Introduction

Intestinal colon cells are polarized epithelial cells that express a wide range of plasma membrane transporters for a variety of substrates. Membrane transporters at the apical border of these cells promote absorption and release of nutrients, electrolytes and water from and to the intestinal lumen. However, membrane transporters at the basolateral border maintain cell homeostasis by the release of these and other nutrients to the interstitium. The apical membrane of intestinal colon cells is directly exposed to agents and toxins, including the enterotoxigenic *Escherichia coli* (ETEC) strains, an intestinal agent leading to diarrhoea in humans [1]. ETEC colonizes host intestines and releases heat-labile and/or heat-stable (STa) enterotoxins. STa causes secretory diarrhoea and is responsible for about half of all ETEC–related diarrhoeal diseases, including traveller’s diarrhoea and epidemic diarrhoea of the newborn [1–5].

STa binds to guanylyl cyclase-C (GC-C) receptors expressed in intestine, kidney, testis and lung, leading to an increase in the intracellular cGMP level [6–8]. STa also increases chloride secretion in a cAMP–dependent manner via the cystic fibrosis transmembrane conductance regulator (CFTR) channels in rat jejunum [9]. In an early study, STa was shown to cause mucosal alkalization due to inhibition of the Na^+^/H^+^ exchange in rat duodenum [10,11]. However, there are not reports addressing whether this enterotoxin modulates intracellular pH (pH_i_), and whether this phenomenon would involve Na^+^/H^+^ exchangers (NHEs) activity. Since both cGMP and cAMP decrease NHEs activity [12,13], an increase in the intracellular pH (pH_i_) in response to STa is expected.

NHEs are key in the modulation of intracellular pH (pH_i_), and are differentially expressed and regulated in intestine epithelial cells [14–17]. At least 11 isoforms of the NHEs family have been identified, out of which NHE1, 2, 3, and 4 are expressed in gastrointestinal membranes [16,17]. NHE4 is highly expressed in the stomach, renal cortex and medulla, ureter, skeletal muscle, heart, liver, and spleen [18]. NHE4 is involved in gastric secretion [19] and plays a large role in controlling pH_i_ [20]. Indeed, NHE4 was identified in the human colon carcinoma cell line T_84_ [21] and in human colonic crypts [13]. This exchanger isoform modulates plays a determinant role in maintaining pH_i_ homeostasis; however, nothing is known about the regulation of NHE4 activity in T_84_ cells by ETEC–released STa. Since T_84_ cells express the GC-C receptors for STa [22], we hypothesize that STa modulates NHE4 activity and the signalling pathways involved in this phenomenon in this cell type. Our findings suggest that STa decreases NHE4 activity, without altering its protein expression via a mechanism that requires cAMP. This could be determinant in the planning of future therapies for human diarrhoea.

## Materials and Methods

### Cell culture

The cell line T_84_ derived from colonic adenocarcinoma of male adult human were purchased from the American Type Culture Collection (ATCC, Rockville, MD, USA) and used for the experiments. T_84_ cells in culture (5% CO_2_, 37°C, pH 7.4) were maintained in Dulbecco’s modified Eagle’s medium F12 (DMEM/F12, Gibco, Grand Island, NY, USA) containing low (5 mmol/L) D-glucose and supplemented with 14.5 mmol/L NaHCO_3_, 3.2 mmol/L D-glutamine, 15 mmol/L HEPES, 5% foetal calf serum (FCS), 100 IU/mL penicillin and 100 mg/mL streptomycin (hereafter referred as primary culture medium (PCM)) as described [21]. Cells were harvested with trypsin/EGTA (0.25/0.2%, 3 minutes, 37°C) and seeded on sterile glass coverslips or 24 well plates for further 72 hours culture until confluence. Cells were then rinsed (3 times) with PCM containing 0.2% FCS (low-FCS/PCM) and cultured in this medium for further 48 hours in order to obtain a cell cycle synchronized culture.

### Measurement of pH_i_

T_84_ cell monolayers in a glass coverslip were mounted in a thermoregulated chamber on an inverted microscope (Nikon Diaphot-TMD, Tokyoi, Japan). The cells were incubated for 10 minutes at 37°C with the fluorescent pH sensitive probe 2,7-bicarboxyethyl-5,6-carboxyfluorescein acetoxymethyl ester (BCECF-AM, 12 μmol/L) (Molecular Probes, Eugene, OR, USA), as described [21]. Cells were then superfused by gravity at 3 mL/minute (37°C) with the control solutions (CS) ((mmol/L) NaCl 141, KCl 5, CaCl_2_ 1, KH_2_PO_4_ 0.4, MgCl_2_ 0.5, MgSO_4_ 0.4, Na_2_HPO_4_ 0.3, HEPES 10, D-glucose 0.6 (pH 7.4, 37°C)) using an electromechanic switching system (Heater and Valve Controller, Yale University Electronics Shop, New Haven, CT, USA). The pH_i_ was calculated from fluorescence ratios measured at excitation of 495/440 nm and emission at 520 nm using a Georgia Instruments PMT-400 photomultiplier system, as described [23]. An area of 260 μm diameter was read, including approximately 200–300 cells. Measurements were performed at 2.5–seconds interval for a period of 300 milliseconds per measurement. The pH_i_ was calibrated using 10 μmol/L nigericin in a calibrating solution ((mmol/L) KCl 130, NaCl 20, CaCl_2_ 1, MgCl_2_ 1, HEPES 5 (pH 6.0, 7.0 and 8.0)) as described [21].

### pH_i_ recovery

The pH_i_ recovery was examined by applying the NH_4_Cl pulse technique [21,23,24]. In brief, BCECF-AM loaded cells were superfused with CS until the basal pH_i_ was stabilized (~15 minutes). T_84_ cells were preincubated with 0.1, 0.25 or 0.75 μmol/L STa for 30 minutes in the presence of 25 μmol/L HOE-694 (a concentration that inhibits NHE1 and NHE2 activity), as described [21,25,26]. The cells were then exposed (2 minutes) to CS supplemented with NH_4_Cl (NH_4_Cl/CS solution) ((mmol/L) NaCl 121, KCl 5.4, CaCl_2_ 1, KH_2_PO_4_ 0.4, MgCl_2_ 0.5, MgSO_4_ 0.4, Na_2_HPO_4_ 0.3, HEPES 10, D-glucose 0.6, NH_4_Cl 20 (pH 7.4, 37°C)). After this incubation period the NH_4_Cl/CS solution was replaced by rinsing the cells with CS free of NH_4_Cl, without or with 25 μmol/L HOE-694, 500 μmol/L sodium nitroprusside (SNP, spontaneous nitric oxide donor) [27], 100 μmol/L dibutyryl cyclic GMP (db-cGMP), 100 nmol/L H89 (a protein kinase A inhibitor)) [28] or 10 μmol/L forskolin (an activator of adenylyl cyclase) [29].

Initial rates of pH_i_ recovery (*dpHi*/*dt*) were calculated from data collected for the first 60 seconds of the recovery (i.e., after removing the NH_4_Cl load) and fitted by a first order lineal regression as described [21,24]. The results were expressed in pH_i_ units/minute. The fraction of *dpHi*/*dt* mediated by NHE4 (^*NHE4*^ *dpHi*/*dt*) was estimated by the expression:

dNHE4pHi/dt=(dTotalpHI/dt) − (dHOEpHi/dt)

where ^*Total*^ *dpHi*/*dt* is the *dpHi*/*dt* estimated in the absence of HOE-694 (i.e., total initial rate), and ^*HOE*^ *dpHi*/*dt* is the *dpHi*/*dt* estimated in the presence of HOE-694, i.e., under inhibition of NHE1 and NHE2 [21]. The relative effect of STa on ^NHE4^ *dpHi*/*dt* (*STa* ^*RE*^) was determined by the expression:

STaRE=100×(dSTa−NHE4pHi/dtdNHE4pHi/dt)

where ^*STa-NHE4*^ *dpHi*/*dt* is ^*NHE4*^ *dpHi*/*dt* measured in the presence of STa.

### Intrinsic buffering capacity

The ability of intrinsic cellular components to buffer changes in pH_i_, i.e., intracellular buffer capacity (ß_i_), was measured as described [21,24]. After determining the basal pH_i_ the cells were incubated in a 0.5 mmol/L KCl-containing Na^+^-free CS (_0_Na^+^/CS) ((mmol/L) N-methyl-D-glucamine (NMDG) 120, KCl 5, CaCl_2_ 1.8, MgCl_2_ 1, HEPES 30, D-glucose 5 (pH 7.4, 37°C)). Cells were then incubated in the latter solution containing decreasing concentrations of NH_4_Cl (50, 20, 10, 5, 2.5 or 1 mmol/L). The ß_i_ (*Beta(i)*) was calculated from the expression:

$$Beta(i)=\frac{\text{change }{\left[NH_{4}^{+}\right]}_{i}}{\text{change }\left(pH_{i}\right)}$$

where the intracellular NH_4_^+^ concentration ([NH_4_^+^]_i_) was obtained from the Henderson-Hasselbalch equation on the assumption that [NH_3_]_i_ (intracellular NH_3_) was equivalent to [NH_3_]_o_ (extracellular NH_3_), and *change (pH*_*i*_ *)* is the fraction of change in units of pH_i_ value. Knowing the *dpHi/dt* and ß_i_ values, the rate of overall transmembrane H^+^ flux (*J*_H_^+^) was calculated from the following expression:

$$J_{H^{+}}=Beta(i)\times \left(\frac{dpHi}{dt}\right)$$

### cAMP and cGMP determination

T_84_ cells were cultured to confluence in 98-well plates. Cells were first treated for 10 minutes with 1 mmol/L 3-isobutyl-1-methylxanthine (IBMX) (Sigma-Aldrich, St. Louis, MO, USA) and next incubated for another 10 minutes with culture medium containing IBMX or IBMX and STa or forskolin. cAMP and cGMP levels were measured by enzyme immunoassay (cAMP or cGMP Direct Biotrak EIA, GE Healthcare, PA, USA) according to manufacturer's instructions. Values of cAMP or cGMP were normalized to total cell protein per well.

### Western blotting

Total protein was obtained from confluent T_84_ cells rinsed (x2) with ice-cold PBS and harvested in 100 μL of lysis buffer (10% SDS, 20% glycerol, 100 mmol/L dithiothreitol, 2.9 mmol/L Tris (pH 6.8), 0.1% bromophenol blue) (63.7 mmol/L Tris/HCl (pH 6.8), 10% glycerol, 2% sodium dodecylsulphate, 1 mmol/L Na_3_VO_4_, 50 mg/mL leupeptin, 5% ß-mercaptoethanol) as described [21,27]. Cells were sonicated (6 cycles, 5 seconds, 100 W, 4°C) and total protein was isolated by centrifugation (13500 *g*, 15 minutes, 4°C). Proteins (50 μg) were separated by polyacrylamide gel (7.5%) electrophoresis, transferred to Immobilon-P polyvinylidene difluoride membranes (BioRad Laboratories, Hertfordshire, UK) and probed with primary monoclonal rabbit *anti*-NHE1 (1:1000 dilution, 12 hours, 4°C), primary polyclonal rabbit *anti*-NHE2 (1:1000 dilution, 12 hours, 4°C) (Abcam, Cambridge, UK), primary rat *anti*-NHE4 antibody (11H11, amino acids 565–675, ~55 kDa) (kindly donated by Dr Daniel Biemesderfer from Yale School of Medicine, New Haven, CT, USA) (1:1000 dilution, 2 hours, 22°C), or monoclonal mouse *anti*-ß-actin (1:5000 dilution, internal reference) (Santa Cruz Biotechnology, Santa Cruz, CA, USA) antibodies. The membranes were rinsed in Tris buffer saline-0.1% Tween 20 (TBS-T) and further incubated (1 hour) in TBS-T/0.2% bovine serum albumin (BSA) containing secondary horseradish peroxidase-conjugated goat *anti*-rat or *anti*-mouse antibodies (Thermo Scientific, Rockford, IL, USA). Proteins were detected by enhanced chemiluminescence (film exposure time was 1 minute) in a ChemiDoc-It 510 Imagen System (UVP, LCC Upland, CA, USA) and quantified by densitometry [27,30].

### Statistical analysis

The values are mean ± S.E.M., where *n* indicates number of different cell cultures (*n* = 27 for STa–untreated (i.e., control) and 25 STa–treated cells) with 3–4 replicates per experiment. The normality of the data (i.e., parametric) was confirmed with Kolmogorov-Smirnov’s test. The variances across the control and STa-treated cells under Bartlett’s test were homogeneous. Comparisons between two groups were performed by means of Student’s unpaired *t*-test. The difference between more than two groups were performed by analysis of variance (ANOVA, one or two-ways). If the ANOVA demonstrated a significant interaction between variables, *post hoc* analyses were performed by the multiple-comparison Bonferroni test. The experimenter running the assays was blinded to the groups allocation before and during the experiments, and when assessing the outcome (i.e., around 30 days). The statistical software GraphPad InStat 3.0b and GraphPad Prism 7.0a.65 (GraphPad Software Inc., San Diego, CA, USA) were used for data analysis. *P*<0.05 was considered statistically significant.

## Results

### Effect of STa on pH_i_ values

**Fig 1:**
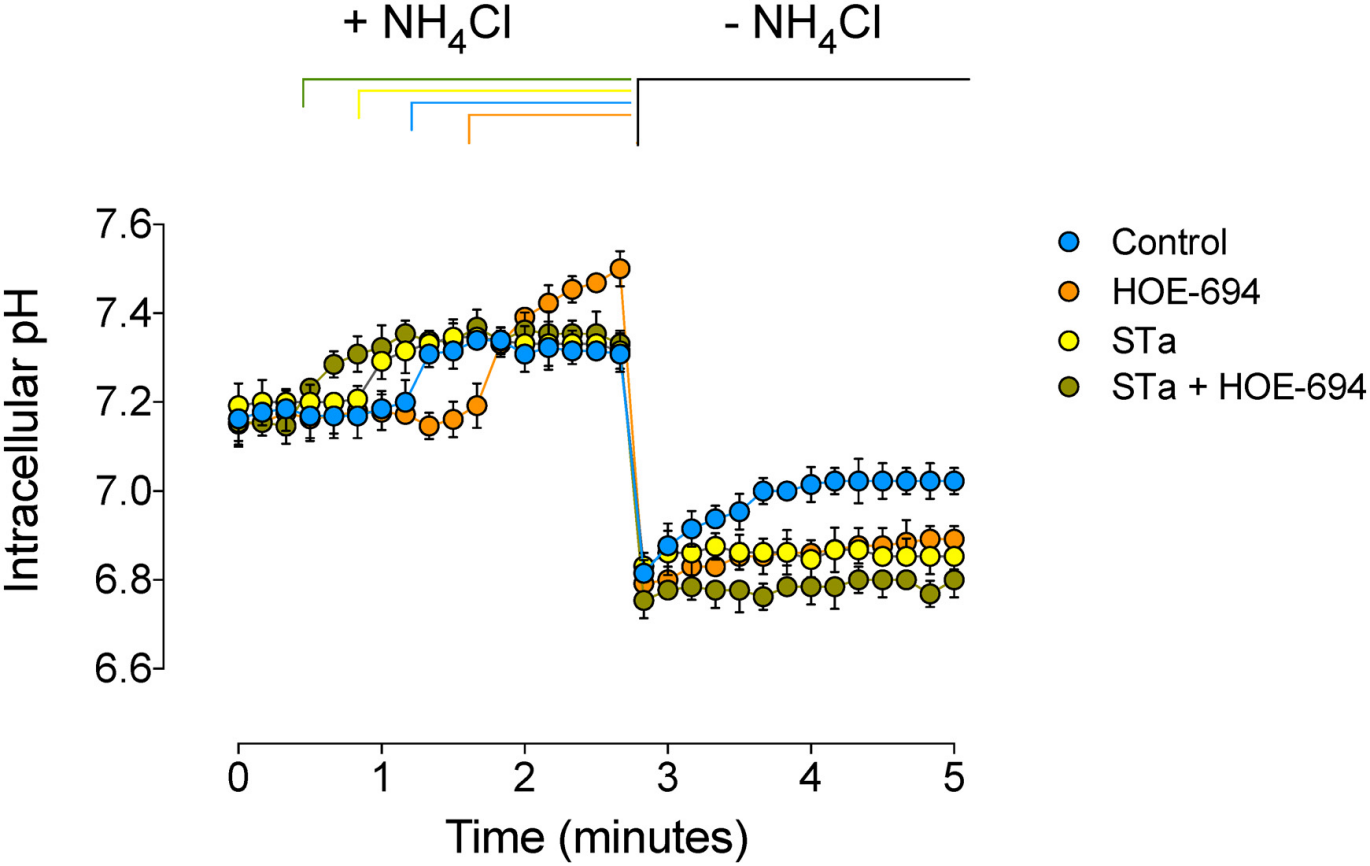
Effect of STa on pH_i_ recovery. T_84_ cells were preloaded with BCECF-AM in the absence or presence (30 minutes) of 0.25 μmol/L heat-stable (STa) enterotoxin. After transferring the cells into a spectrofluorometer the basal pH_i_ was stabilized and then exposed (1.5–2 minutes) to a control solution containing 20 mmol/L NH_4_Cl (+ NH_4_Cl). Cells were then rinsed with a NH_4_Cl–free solution (–NH_4_Cl) and left in this medium without (Control) or with 0.25 μmol/L STa, 25 μmol/L HOE-694, or both (STa + HOE-694) (see Methods). Initial rates of pH_i_ recovery were calculated from data collected for the first 60 seconds after removing the NH_4_Cl load. Values are mean ± S.E.M. (*n* = 25–27).

Basal pH_i_ in T_84_ cells detected in this study was comparable to previous reports in this cell type [21,31,32] and was unaltered in cells preincubated with STa (Table 1, Fig 1). Following the NH_4_Cl pulse the acidic pH_i_ values detected in the cells exposed to STa or HOE-694 were partially restored (27 ± 3 or 55 ± 6%, respectively) compared with cells in the absence of these agents (Fig 1). When cells were coincubated with STa + HOE-694 the NH_4_Cl–induced acidic pH_i_ was only minimally restored (9 ± 1%).

**Table 1:** Modulation of intracellular pH by STa in T_84_ cells.

	*pHi*	*dpHi/dt*
Control	7.170 ± 0.028	0.133 ± 0.009
STa	7.144 ± 0.019	0.046 ± 0.009 *
HOE-694	7.172 ± 0.034	0.051 ± 0.010 *
HOE-694 + STa	7.130 ± 0.046	0.014 ± 0.001 * †
Forskolin	7.156 ± 0.021	0.051 ± 0.010 *
Forskolin + STa	7.171 ± 0.030	0.048 ± 0.009 *
Forskolin + HOE-694	7.161 ± 0.050	0.017 ± 0.003 * ‡
Forskolin + HOE-694 + STa	7.125 ± 0.061	0.016 ± 0.002 * ‡
H89 + HOE-694 + STa	7.143 ± 0.038	0.046 ± 0.008 *
db-cGMP	7.21 ± 0.054	0.110 ± 0.012
db-cGMP + STa	7.10 ± 0.021	0.050 ± 0.012 * §
db-cGMP + HOE-694	7.19 ± 0.053	0.057 ± 0.002 * §
db-cGMP + HOE-694 + STa	7.14 ± 0.051	0.015 ± 0.001 * § ¶
SNP	7.16 ± 0.026	0.123 ± 0.009
SNP + STa	7.15 ± 0.021	0.045 ± 0.011 *&
SNP + HOE-694	7.11 ± 0.024	0.047 ± 0.009 *&
SNP + HOE-694 + STa	7.14 ± 0.052	0.015 ± 0.012 *&$

**Fig 2:**
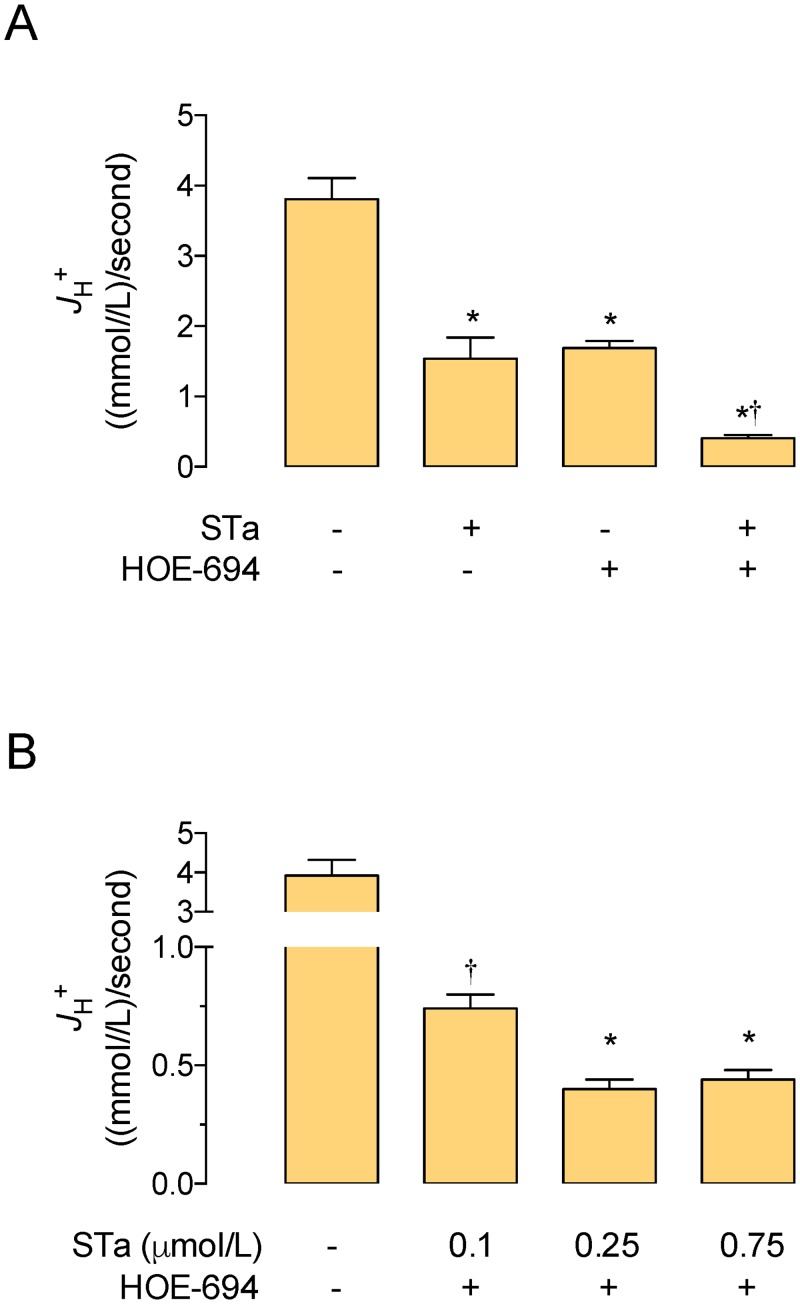
Effect of STa on *J*_H+_. The overall transmembrane H^+^ flux rates (*J*_H+_) were calculated from initial rates of pH_i_ recovery and the intrinsic buffer capacity (*βi*) values (see Methods). **A**, T_84_ cells were exposed to culture medium without (–, Control) or with (+) 0.25 μmol/L heat-stable (STa) enterotoxin, 25 μmol/L HOE-694, or both (see Methods). **B**, T_84_ cells were exposed to increasing concentrations of STa in the presence of 25 μmol/L HOE-694 as in A. In A, *P<0.05 versus Control, †*P*<0.05 versus STa or HOE-694. In B, *P<0.05 versus Control, †*P*<0.05 versus other values in STa + HOE-694. Values are mean ± S.E.M. (*n* = 25–27).

The intracellular pH (*pHi*) was measured in BCECF-AM–preloaded T_84_ cells as described in Methods. Cells were also subjected to an acid pulse (NH_4_Cl assay) and the initial rates of pH_i_ recovery (*dpHi*/*dt*) was measured in cells in the absence (Control) or presence (30 minutes) of 0.25 μmol/L heat-stable (STa) enterotoxin, 25 μmol/L HOE-694 (Na^+^/H^+^ exchangers inhibitor), 10 μmol/L forskolin, 100 nmol/L H89 (protein kinase A inhibitor), 100 μmol/L dibutyryl cyclic GMP (db-cGMP), or 500 μmol/L sodium nitroprusside (SNP). STa at 0.1 and 0.75 μmol/L did not alter pHi values (7.121 ± 0.011 and 7.160 ± 0.014, respectively; *P*>0.05, *n* = 4). STa at 0.1 μmol/L partially reduced *dpHi*/*dt* value (0.098 ± 0.005 pHi units/minute, *P*<0.05, *n* = 4), and inhibition at 0.75 μmol/L (0.056 ± 0.007 pHi units/minute) was similar (*P*>0.05, *n* = 4) to 0.25 μmol/L STa (see also Fig 2B).

*P<0.04 versus Control

^†^ *P*<0.03 versus STa or HOE-694

^‡^ *P*<0.03 versus Forskolin, Forskolin + STa, and H89 + HOE-694 + STa,

^§^ *P*<0.05 versus db-cGMP

^¶^ *P*<0.05 versus db-cGMP + STa and db-cGMP + HOE-694, &*P*<0.05 versus db-cGMP

^$^ *P*<0.03 versus SNP + STa and SNP + HOE-694

### Effect of STa on pH_i_ recovery kinetics

Since T_84_ cells express NHE1, NHE2 and NHE4, but not NHE3 forms [21,33], we assayed which of these forms was involved in STa effect on *dpHi*/*dt*. The *dpHi*/*dt* values in the presence of STa or HOE-694 were lower (65 ± 7 or 62 ± 6%, respectively) when compared with cells in the absence of these molecules (Table 1). Coincubation of cells with STa + HOE-694 resulted in higher reduction (90 ± 6%) in the *dpHi*/*dt* compared with the reduction seen in cells treated with STa or HOE-694 alone.

### Effect of STa on ß_i_ and *J*_H_^+^

The ß_i_ value detected in T_84_ cells in the absence of STa (31.1 ± 2.5 (mmol/L)/ intracellular pH units) was similar to that previously reported for this cell type under the same culture and measurement conditions (~31 (mmol/L)/intracellular pH units) [21]. Change in ß_i_ value was not significantly altered by 0.25 μmol/L STa in a range of 1.6 pH_i_ units in T_84_ cells. Parallel assays show that cells treated with STa exhibit decreased *J*_H_^+^ (60 ± 7%) compared with cells in the absence of this toxin (Fig 2A). Since maximal inhibitory effect on this parameter was achieved with 0.25 μmol/L STa in the presence of 25 μmol/L HOE-694 (Fig 2B), this concentration was used in all subsequent experiments. HOE-694 caused a decrease in *J*_H_^+^ (56 ± 7%) that was similar (*P*>0.05) to that in cells in the presence of STa. Coincubation of cells with STa + HOE-694 resulted in a decrease in *J*_H_^+^ (89 ± 6%) that was higher compared with the effect seen in cells treated with STa or HOE-694 alone.

### NHE1, NHE2 and NHE4 protein abundance

**Fig 3:**
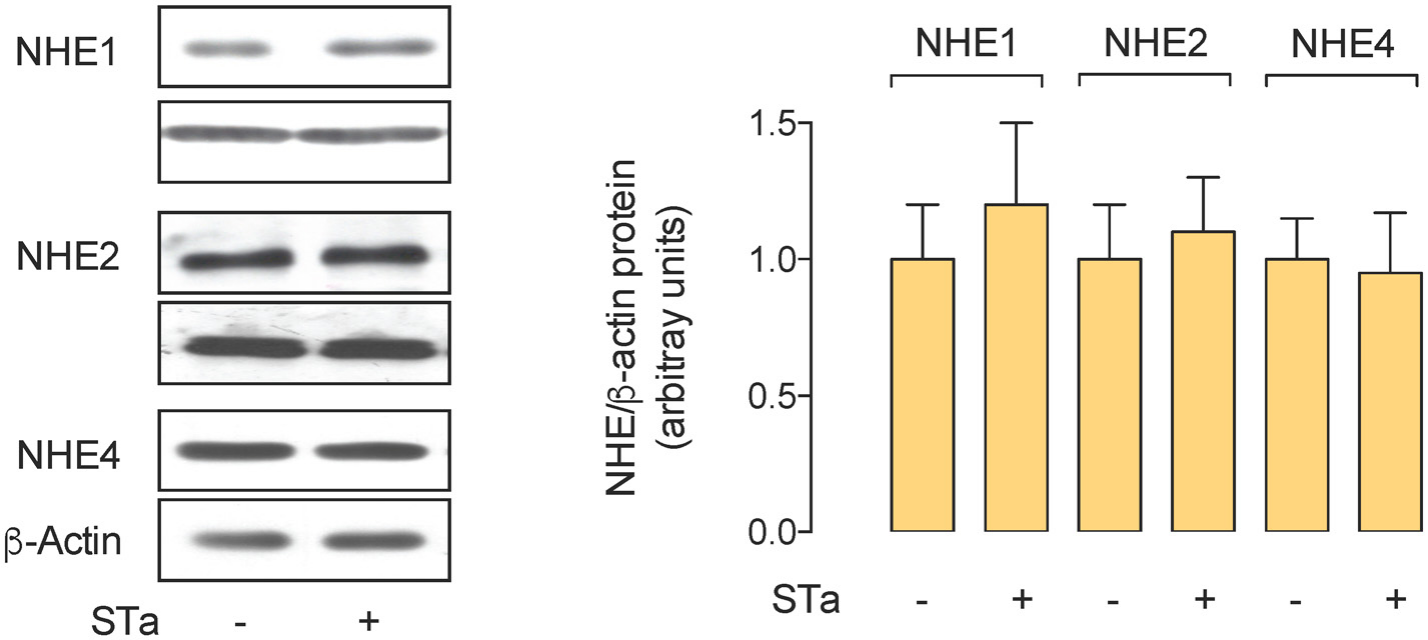
Effect of STa on NHE4 protein abundance. Western blot for NHE4 protein abundance in whole extracts of T_84_ cells exposed for 30 minutes in the absence (Control) or presence (STa) of 0.25 μmol/L heat-stable (STa) enterotoxin. *Lower panel*: NHE4/β-actin ratio densitometries normalized to 1 in Control. β-Actin is internal reference. Values are mean ± S.E.M. (*n* = 15).

To address whether STa–associated decrease in *J*_H_^+^ was due to lower protein abundance of NHE4, or whether this toxin alters NHE1 or NHE2 protein abundance, the protein level of these membrane transporters was assayed. The results show that incubation of T_84_ cells with STa did not alter NHE1, NHE2 or NHE4 protein abundance (Fig 3).

### cGMP and cAMP involvement on NHE4–mediated pH_i_ recovery kinetics

**Fig 4:**
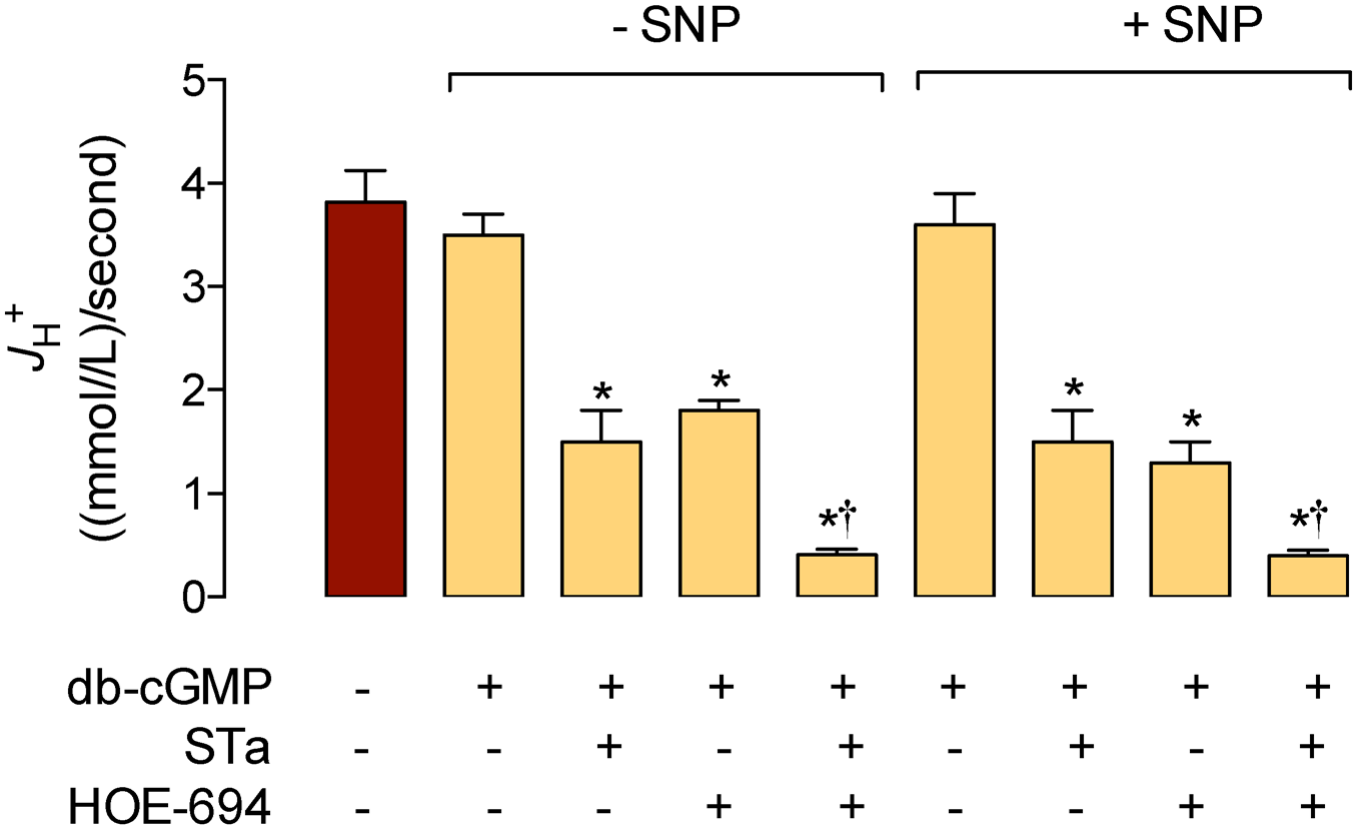
Involvement of cGMP on STa modulation of *J*_H+_. T_84_ cells were exposed for 30 minutes in the absence (–SNP) or presence (+ SNP) of 500 μmol/L sodium nitroprusside (SNP). The overall transmembrane H^+^ flux rates (*J*_H+_) were calculated from initial rates of pH_i_ recovery and the intrinsic buffer capacity (*βi*) values (see Methods). Cells were exposed to culture medium without (–, Control, red bar) or with (+) 100 μmol/L dibutyryl cyclic GMP (db-cGMP), 0.25 μmol/L STa, and/or 25 μmol/L HOE-694 (see Methods). *P<0.05 versus Control or corresponding db-CGMP, †*P*<0.05 versus corresponding STa or HOE-694 in the presence of db-cGMP. Values are mean ± S.E.M. (*n* = 25–27).

STa is shown to increase the cGMP level in T_84_ cells [34]; however, the role of cGMP as modulator of NHE4 activity is not addressed [17]. Thus, we next investigated whether STa effect on NHE4–dependent *dpHi*/*dt* in this cell type was modulated by direct administration of exogenous cGMP. The results show that *dpHi*/*dt* and basal pH_i_ (Table 1), and *J*_H_^+^ (Fig 4) were unaltered in T_84_ cells exposed to db-cGMP in the absence of HOE-694 or STa. However, the reduction in *dpHi*/*dt* and *J*_H_^+^ seen in response to STa, HOE-694, or STa + HOE-694 was unaltered by db-cGMP. When cells were incubated with SNP (a spontaneous NO donor) [27] the results were similar to those in the presence of db-cGMP (Table 1, Fig 4). Parallel results show that cGMP intracellular level was increased by STa and SNP, confirming previous reports in T_84_ cells [35] and rat distal colon crypts [36], but it was unaltered by HOE-694 (not shown).

**Fig 5:**
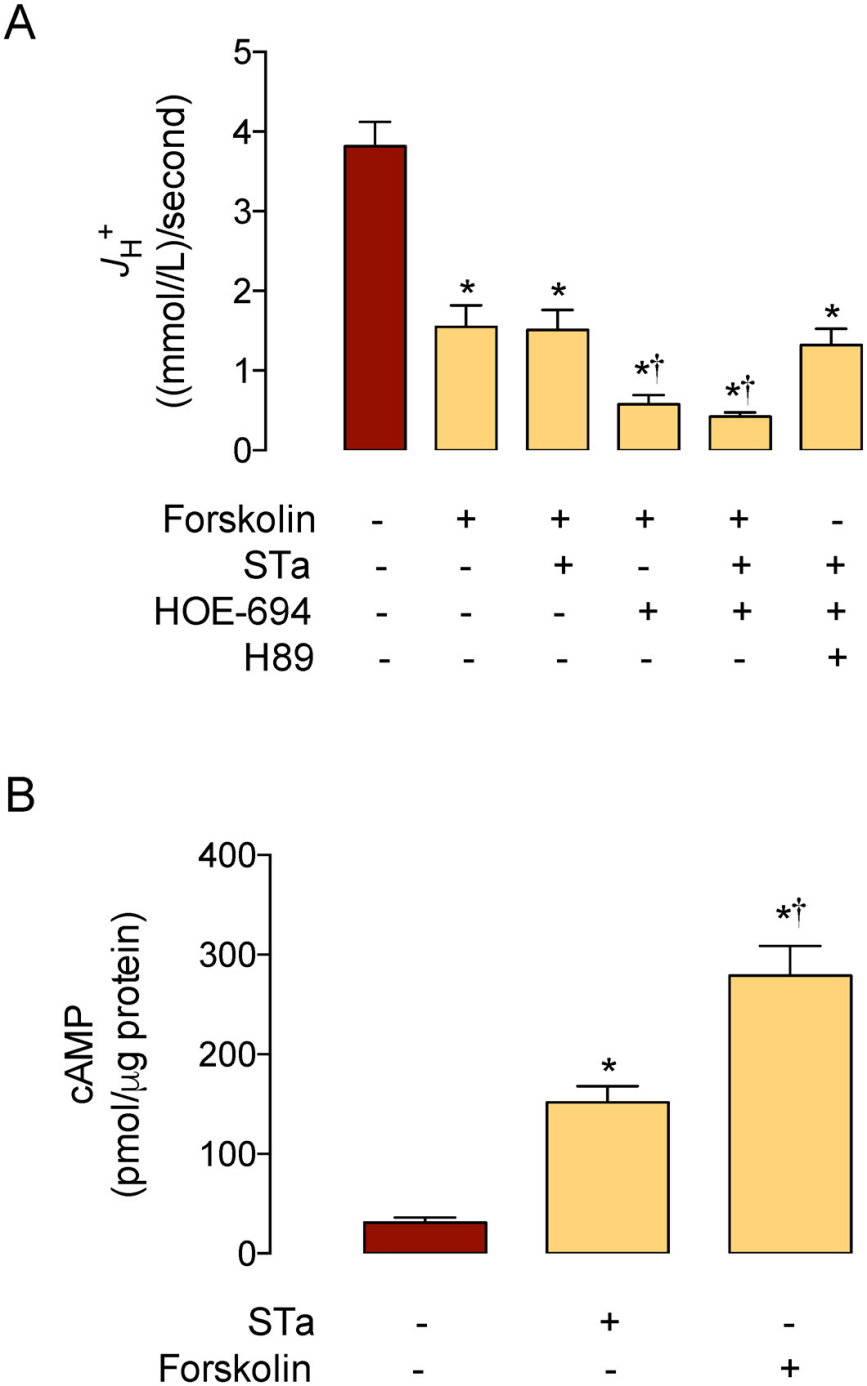
Involvement of cAMP and PKA on STa modulation of *J*_H+_. A, The overall transmembrane H^+^ flux rates (*J*_H+_) were calculated from initial rates of pH_i_ recovery and the intrinsic buffer capacity (*βi*) values (see Methods). Cells were exposed to culture medium without (–, Control, red bar) or with (+) 10 μmol/L forskolin, 0.25 μmol/L heat-stable (STa) enterotoxin, 25 μmol/L HOE-694, and/or 100 nmol/L H89 (see Methods). B, cAMP levels in cells in the absence (Control) or presence of STa or forskolin as in A. In A, *P<0.05 versus Control, †*P*<0.05 versus STa, HOE-694, or STa + HOE-694. In B, *P<0.05 versus Control, †*P*<0.05 versus STa. Values are mean ± S.E.M. (*n* = 25–27).

We next assayed whether cAMP was involved in the response of T_84_ cells to STa–reduced NHE4–mediated pH_i_ recovery kinetics. Cells incubated with forskolin (adenylyl cyclase activator) [29] in the absence of HOE-694 resulted in a decrease in *dpHi*/*dt* (Table 1) and *J*_H_^+^ (Fig 5A) that was of a similar magnitude to the decrease seen in cells incubated with STa in the absence or presence of this activator. However, in the presence of HOE-694 or STa + HOE-694, forskolin caused a reduction in these parameters that was similar to that seen in cells coincubated with STa + HOE-694 in the absence of this activator. Parallel results show that intracellular level of cAMP increased by STa (4.9 ± 0.5 fold) and forskolin (8.9 ± 1.5 fold) (Fig 5B). Additionally, preincubation of cells with H89 (inhibitor of PKA) [28] reversed the decrease in *dpHi*/*dt* and *J*_H_^+^ caused by STa + HOE-694 to values that are comparable to STa or HOE-694 alone.

## Discussion

This study shows that the enterotoxigenic *Escherichia coli* (ETEC) released heat-stable (STa) enterotoxin decreases the pH_i_ recovery kinetics in the human colocarcinoma T_84_ cell line. This phenomenon results from a lower activity of NHE4 without altering its protein expression. STa effect depends on the level of cAMP, but not cGMP, and PKA activation. These findings represent a novel mechanism of pH_i_ homeostasis by STa that could have consequences in the physiology of gastrointestinal cells leading to human diarrhoea.

### STa modulation of NHEs activity

STa is an enterotoxin that causes gastrointestinal electrolyte imbalance characterized by a higher Cl^-^release to the gastrointestinal lumen, a phenomenon that ends in diarrhoea in humans [1,3–5]. One of the potential mechanisms for these adverse effects of STa is a mucosal alkalization due to lower activity of plasma membrane mechanisms involved in maintaining transmembrane distribution of H^+^, including NHEs activity [10,11]. Our results show that STa caused a decrease in NHEs activity resulting in lower H^+^ efflux (i.e., *J*_H_^+^). This phenomenon may be responsible for the observed reduction in the capacity to restore the pH_i_ recovery kinetics after an acid pulse. This possibility is supported by the findings showing that STa caused a similar reduction in *dpHi*/*dt* and *J*_H_^+^ (reduction in *dpHi*/*dt* / reduction in *J*_H_^+^ = 1.1), thus, making possible that alterations in the pH_i_ recovery rate caused by STa was due to reduced H^+^ efflux kinetics. In addition, since the intrinsic buffering capacity (ß_i_) values were unaltered by STa (ß_i_ with STa/ß_i_ without STa = 1), it is unlikely that these alterations were the result of an altered ß_i_ in T_84_ cells. Indeed, in cells incubated with STa the pH_i_ value was not significantly altered (pH_i_ with STa/ pH_i_ without STa = 0.996) compared with cells in the absence of this toxin.

Interestingly, it was initially shown [31] that T_84_ cells express mainly NHEs (NHE1, NHE2 and NHE4) [21], in a minor grade Cl^-^/HCO_3_^-^ exchangers and Na^+^/HCO_3_^-^ cotransporters, but not other classical mechanisms of H^+^ export such as the vacuolar H^+^-ATPases [37] or H^+^/K^+^-ATPases [38]. Out of these membrane transport systems, NHEs play a major role in the removal of intracellular H^+^ in most cell types maintaining stable pH_i_ and extracellular pH values [14–17,20,37,38].

NHE4 is an isoform of the NHEs family of membrane exchangers whose function results in the modulation of pH_i_ in mammalian cells [14,16,17]. This membrane Na^+^/H^+^ exchanger isoform is expressed in the human gastrointestinal tract, and is co-expressed with NHE1 and NHE2, but not NHE3, in T_84_ cells [21,33], as confirmed in this study. Interestingly, cells exposed to HOE-694 show lower *dpHi*/*dt* and *J*_H_^+^ most likely via a mechanism involving lower activity of NHE1 and NHE2 isoforms, since the concentration of this inhibitor used in the present study (25 μmol/L) preferentially inhibits these isoforms, but not NHE4 [21,26]. Indeed, cells in the presence of HOE-694 show partial recovery of the pH_i_ value suggesting that not all the pH_i_ recovery is mediated by NHE1 and NHE2, but other mechanism(s) is plausible in this cell type.

Since STa in the presence of HOE-694, i.e., where NHE1 and NHE2 were not functional, almost abolished the *dpHi*/*dt* and *J*_H_^+^ (both reduced by ~90%), it is likely that NHE4 isoform was inhibited by this enterotoxin in T_84_ cells. This possibility is supported when we consider that the concentration of STa used in our study is close to the STa half-maximal stimulatory concentration for cGMP accumulation reported in T_84_ cells [25]. Additionally, the possibility that STa reduces the *dpHi*/*dt* and *J*_H_^+^ via a mechanism including lower expression of NHE4, or NHE1 or NHE2, is unlikely since the protein abundance for none of these isoforms were altered by the toxin. Thus, STa–reduced H^+^ efflux seems to be due to a lower activity rather than expression of NHE4 in this cell type. STa effect in the presence of HOE-694 leads a remaining fraction of pH_i_ recovery that accounted for 10% of the total recovery after an acid pulse. This finding could results from other mechanisms than inhibition of NHE1, 2 or 4, such as activity of Cl^-^/HCO_3_^-^ exchangers and/or Na^+^/HCO_3_^-^ cotransporters expressed in T_84_ cells [31]. Indeed, STa was shown to increase HCO_3_^-^ secretion via a higher Na^+^/HCO_3_^-^ activity in duodenal CFRT^–/–^mice [39]. However, our pH_i_ recovery assays were performed in the absence of extracellular HCO_3_^-^ in this cell type making the latter unlikely.

### Involvement of cAMP on STa effect

It has been shown that STa increases Cl^-^secretion in a cAMP–and cGMP–dependent manner via CFTR channels in rat jejunum [9]. Initial reports show that STa–increased cGMP, but unaltered cAMP level in rabbit distal ileum mucosa [40] or reduced cAMP level in mice intestine [41]. Our results show that exposure of T_84_ cells to STa results in increased cGMP and cAMP levels. Since these nucleotides decrease NHEs activity [12,13], STa–increased levels may have functional consequences on pH_i_ recovery in T_84_ cells.

Since incubation of cells with exogenous cGMP (db-cGMP) did not alter basal *dpHi*/*dt* and *J*_H_^+^ in our assays it is likely that this cyclic nucleotide is not involved in the modulation of NHEs activity in T_84_ cells. Furthermore, the inhibitory effect of STa on *dpHi*/*dt* and *J*_H_^+^ in the presence of HOE-694 was unaltered by db-cGMP, suggesting that NHE4 inhibition by STa was independent of cGMP. This is supported by the findings showing that *dpHi*/*dt* and *J*_H_^+^ inhibition by STa or HOE-694 alone was unaltered when cells were coincubated with these molecules and db-cGMP. Additionally, exposure of cells to exogenous NO delivered by SNP, a spontaneous NO donor [27], does not change STa effect in the absence or presence of HOE-694. Since SNP did not alter the reduction in the *dpHi*/*dt* and *J*_H_^+^ caused by HOE-694 itself, NO in this cell type may not alter this inhibitors’ effectiveness on NHE1 and NHE2.

It was early shown that forskolin, a potent activator of adenylyl cyclase, has a profound effect in T_84_ transmonolayer net water flux (*J*_W_) [29], suggesting that cAMP could be involved in this phenomenon. Unfortunately, the cAMP level was not determined in the latter study. Additionally, incubation of T_84_ cells with secretagogues whose actions are mediated by cAMP ends with Cl^-^secretion from this cell type [35,42–44]. However, it is paradoxical that even when the level of cAMP was found unaltered in T_84_ cells in response to STa, this toxin effect on Cl^-^secretion closely resembles a cAMP–mediated mechanism in this cell type [35]. Our findings show that cAMP level is increased in T_84_ cells treated with STa or with forskolin. Since the effect of forskolin alone was to diminish the *dpHi*/*dt* and *J*_H_^+^ in a same magnitude as STa alone or STa + forskolin, it is likely that a higher cAMP level could be involved in downregulation of NHE4 activity in this cell type. Parallel results suggest that NHE1 and NHE2 may not be under modulation by STa–or forskolin–mediated cAMP increase since the inhibition caused by HOE-694 of *dpHi*/*dt* and *J*_H_^+^ by itself or in the presence of STa was unaltered by forskolin. Interestingly, since H89, a PKA inhibitor, resulted in restoration of the reduced *dpHi*/*dt* and *J*_H_^+^ seen in the presence of STa + HOE-694 + forskolin to values that are comparable to those in the presence of these molecules per separate, it is likely that PKA may mediate STa inhibition of NHE4 in T_84_ cells.

**Fig 6:**
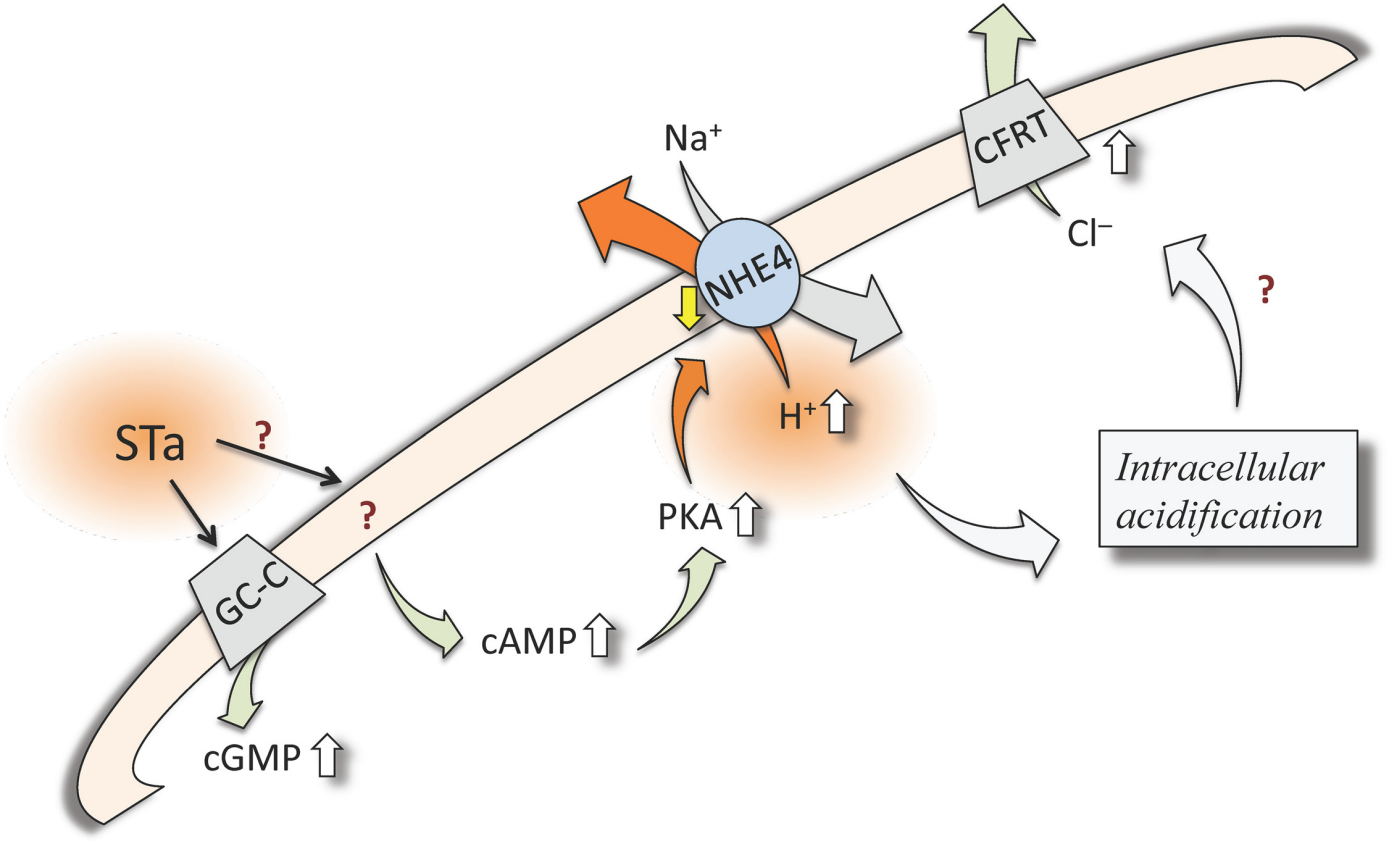
Potential involvement of cAMP and PKA on STa modulation of *J*_H+_. In T_84_ cells the enterotoxigenic *Escherichia coli* (ETEC) released heat-stable enterotoxin (STa) activates guanylyl cyclase-C (GC-C) receptors to generate (green arrow) cyclic GMP (cGMP) increasing (⇧) its intracellular level. STa also increases cyclic AMP (cAMP) level via a mechanism that is not well defined in this cell type (?). Increase in cAMP activates protein kinase A (PKA), which could be responsible of a reduced (⇩) activity of the Na^+^/H^+^ exchanger isoform 4 (NHE4). The resulting intracellular accumulation of H+ leads to intracellular acidification, a phenomenon that, via undefined mechanism, could be responsible for the increase in chloride (Cl^-^) secretion via the cystic fibrosis transmembrane conductance regulator channels (CFRT) reported in this cell type and human diarrhoea.

In conclusion, the enterotoxigenic *Escherichia coli* released STa enterotoxin has a deleterious effect on the normal physiology of T_84_ cells *in vitro*. In terms of its association with human diarrhoea this enterotoxin was found to increase not only cGMP levels, but also the cAMP level, perhaps leading to PKA activation in this cell type. It is proposed that STa reduces the capacity of T_84_ cells to recover the pH_i_ after an acid pulse via a mechanism that includes reduced activity of NHE4, but not NHE1 or NHE2, in this cell type. These findings constitute a novel mechanism of pH_i_ homeostasis by STa in this cell type, and perhaps in the gastrointestinal epithelium, resulting in a deficient recovery rate and H^+^ efflux after metabolic alterations associated with intracellular acidification. These findings complement the reduced transepithelial electrical resistance caused by STa in T_84_ cells, indicative of an intestinal barrier dysfunction in addition to STa–induced water secretion [45]. Considering that T_84_ cells respond with increased Cl^-^release to STa via cGMP–and cAMP–dependent mechanisms, a role of NHE4 is this phenomenon is proposed. All together the alterations caused by STa in a functional sequence (i.e., STa / increased cAMP / increased PKA activity / decreased NHE4 activity / increased intracellular acidification) (Fig 6) could have consequences in the physiology of gastrointestinal cells promoting human diarrhoea.
